# Profiles of theory of mind impairments and personality in clinical and community samples: integrating the alternative DSM-5 model for personality disorders

**DOI:** 10.3389/fpsyt.2023.1292680

**Published:** 2024-01-11

**Authors:** Mireille Lampron, Amélie M. Achim, Dominick Gamache, Allyson Bernier, Stéphane Sabourin, Claudia Savard

**Affiliations:** ^1^School of Psychology, Université Laval, Québec City, QC, Canada; ^2^CERVO Brain Research Centre, Québec City, QC, Canada; ^3^Department of Psychiatry and Neurosciences, Université Laval, Québec City, QC, Canada; ^4^VITAM – Centre de recherche en santé durable, Québec City, QC, Canada; ^5^Department of Psychology, Université du Québec à Trois-Rivières, Québec City, QC, Canada; ^6^Department of Educational Fundamentals and Practices, Université Laval, Québec City, QC, Canada

**Keywords:** theory of mind, personality disorders, DSM-5 alternative model for personality disorders, personality functioning, personality traits

## Abstract

**Introduction:**

Deficits in theory of mind (ToM)—the ability to infer the mental states of others—have been linked to antagonistic traits in community samples. ToM deficits have also been identified in people with personality disorders (PD), although with conflicting evidence, partly due to the use of categorical diagnoses. The DSM-5 Alternative Model for Personality Disorders (AMPD) provides an opportunity for a more precise understanding of the interplay between ToM abilities and personality pathology. Therefore, the study aims to determine whether and how individuals with diverse ToM profiles differ regarding personality impairment (AMPD Criterion A) and pathological facets (AMPD Criterion B).

**Method:**

Adults with PD (*n* = 39) and from the community (*n* = 42) completed tests assessing ToM skills and self-reported questionnaires assessing AMPD Criteria A and B. Hierarchical agglomerative and TwoStep cluster analyses were consecutively computed using scores and subscores from ToM tests as clustering variables. Multivariate analyses of variance were subsequently performed to compare the clusters on both AMPD Criteria. Five clinically and conceptually meaningful clusters were found. The most notable differences across clusters were observed for Intimacy and Empathy dysfunctions (Criterion A), as well as for the Deceitfulness, Callousness, and Hostility facets from the Antagonism domain and the Restricted affectivity facet from the Detachment domain (Criterion B).

**Discussion:**

The results support the association between antagonistic personality facets and ToM deficits. However, clusters showing impairments in ToM abilities did not necessarily exhibit high levels of personality dysfunction or pathological facets, emphasizing that both constructs are not isomorphic. Nevertheless, specific profiles can help refine existing interventions to make them more sensitive and specific to the nature of ToM dysfunctions while considering personality functioning and facets.

## Introduction

Theory of mind (ToM) is a social cognitive ability enabling individuals to appreciate the mental states of others, such as beliefs, emotions, or intentions, and to recognize that other people’s mental states may be different from their own (1). Adequate ToM skills are critical to the development of satisfying interpersonal relationships or effective communication, and ToM deficits can lead to poor functional outcomes, lower quality of life, or diminished well-being (2, 3). ToM skills vary from one individual to another, notably according to their personality, which has been demonstrated in both individuals with personality disorders (PD) and from the community (4).

Using the widely validated Five-Factor Model in community samples, ToM abilities have been positively associated with Agreeableness, a tendency to be prosocial, trustworthy, altruistic, or cooperative (5–7). Moreover, antagonistic personality traits such as Machiavellianism, psychopathy or narcissism, characterized by a low level of Agreeableness (8), have consistently been associated with a reduced capacity to infer mental states in community samples (9–13). Less frequent associations have been found with Neuroticism—a propensity to experience negative emotions such as depression, anxiety, and anger (7).

ToM abilities also proved to be altered in many disorders featuring substantial difficulties in interpersonal relationships, such as schizophrenia, autism spectrum disorders, or—although more inconsistently—personality disorders (PD; 14). Results from the available studies, mainly focused on borderline and antisocial PDs, are discordant, and consensus about the presence or nature of ToM deficits in the PD population remains elusive (15). The variety of ToM tasks used in previous studies may explain these discrepancies. In fact, ToM is not a unidimensional construct but instead includes different, interdependent processes that distinctly affect behavior or brain activation (16, 17). Thus, different tasks may measure specific ToM processes or require distinct cognitive processes, which may, in turn, influence performance (18).

The utilization of categorical diagnostic models, which fail to adequately represent the structure of PD pathology (19), may also explain the mixed results found in the ToM literature for the PD population. In fact, many authors found that ToM dysfunctions may be linked to the personality style or the severity of personality impairments rather than specific diagnoses (20, 21).

The shortcomings of the long-established categorical model [see (22) for a review] have led to a shift toward dimensional models for PD in the *International Classification of Diseases and Related Health Problems*, 11th Revision (ICD-11; 23) and the fifth edition of the *Diagnostic and Statistical Manual of Mental Disorders* (DSM-5). The Alternative Model for Personality Disorders (AMPD) in Section III (Emerging Measures and Models; 24) was introduced in the latter. The AMPD is based on two criteria. Criterion A pertains to four elements assessing the severity of personality dysfunction (from *little or no impairment* to *extreme impairment*) based on self (Identity and Self-direction) and interpersonal (Intimacy and Empathy) functioning, with Empathy being closely related to ToM from a conceptual standpoint (24; See Supplementary Table 1 for definitions). Criterion B consists of 25 maladaptive personality facets organized into five broader domains: Negative Affectivity, Detachment, Antagonism, Disinhibition, and Psychoticism (24; See Supplementary Table 1 for definitions). The introduction of the AMPD provides an opportunity for a more fine-grained understanding of the interplay between ToM abilities and PD pathology by allowing the coverage of a wide range of severity and a large diversity of pathological personality traits, thus facilitating the integration of results from both clinical and community participants. Therefore, using the AMPD would permit the recruitment of participants typically excluded from previous studies, such as those with mixed PD and people with personality disturbances that do not reach the severity criteria for a PD diagnosis (15).

Despite researchers’ enthusiasm for this new model, which resulted in considerable research since its introduction, very few studies have investigated the link between the AMPD and ToM. Fossati et al. (25) assessed the relationship between ToM and AMPD’s Criterion B in a sample of PD outpatients and inpatients on two different tests measuring ToM (i.e., the *Movie for the Assessment of Social Cognition* and the *Reading the Mind in the Eyes test* [RMET]). Most of the significant correlations found between ToM tests and personality traits in that study were with facets from the Antagonism and Detachment domains. In the same study, some significant correlations were also found between ToM tests and a limited number of facets from the Negative Affectivity (Emotional lability), Disinhibition (Lack of rigid perfectionism and Risk-taking), and Psychoticism (Unusual beliefs and experiences) domains.

A study by da Costa et al. (26) in a community sample also found ToM difficulties in participants with higher levels of antagonistic personality traits. Furthermore, women with higher levels of Negative Affectivity or lower levels of Detachment were better at identifying positive emotions depicted in pictures of the eyes. Finally, Hanegraaf et al. (4) explored social processing (including the ability to perceive, understand, and respond to others) in subgroups created from the five AMPD domains in a community sample. They highlighted social processing impairments in a cluster with a high level of Antagonism and Disinhibition.

In summary, investigating the interconnections between ToM and personality as conceptualized with the AMPD may help obtain more straightforward, consistent, and precise results in both PD and community samples. A better insight into the interaction between personality and ToM could also help understand relational difficulties in people with pathological personality or ToM deficits, leading to more targeted interventions. However, studies examining ToM and personality with the AMPD are sparse, and none have assessed Criterion A. The person-centered approach adopted in the present study on the relationships between ToM deficits and personality pathology will provide initial data on how these key areas can co-occur in individuals, potentially highlighting different profiles of interrelations. Specifically, the study aims to use tasks assessing ToM to create clusters based on ToM abilities and to explore if individuals from different clusters might differ regarding their personality functioning (Criterion A) and their pathological traits (Criterion B).

Considering the strong association between ToM and interpersonal relationships, it is expected that more severe impairments on the interpersonal functioning elements of Criterion A (Intimacy and Empathy) would be found in subgroups characterized by more severe ToM impairments. Based on the previous studies by Fossati et al. (25), da Costa et al. (26), and Hanegraaf et al. (4), it is expected that higher levels of Antagonistic and Detachment personality facets would characterize subgroups presenting ToM impairments. Finally, since schizotypal personality disorder is considered a milder expression of schizophrenia spectrum disorders (27), a population in which ToM deficits are often observed (28), it is expected that the traits forming the schizotypal PD, i.e., Unusual beliefs and experiences, Eccentricity, and Cognitive and perceptual dysregulation from the Psychoticism domain, in addition to Restricted affectivity, Withdrawal, and Suspiciousness from the Detachment domain, will be more elevated in clusters with poorer ToM performance (24).

## Materials and methods

### Sample

Participants from the community were recruited through posters in local stores, social media, or emails sent to students and employees at Université Laval. Participants with PD were recruited through institutions from the Centre intégré universitaire de santé et de services sociaux de la Capitale-Nationale, offering general or specialized treatments to individuals with PD. They were recruited using posters displayed in waiting areas. Members of the research team also presented the project during group therapy sessions.

For both groups, all those interested in the study were invited to contact the research team for more information. In total, 92 participants expressed an interest in pursuing the process. Of those 92 participants, 81 corresponded to our inclusion criteria and were thus retained for the study. Eleven participants were excluded, notably because they reported a neurological disorder (*n* = 6), a traumatic brain injury (*n* = 4), or because they did not speak French as their first language (having attended school in French was accepted; *n* = 1). The occurrence of a severe substance-related and addictive disorder or an intellectual disability, i.e., estimated IQ under 70, were also exclusion criteria. Still, no participants were ruled out for this reason. However, since they are frequent comorbid conditions with PD, well-controlled attention deficit hyperactivity disorder, mild substance-related and addictive disorder, or traumatic brain injury with no lasting effects (29–33) were tolerated to preserve the representativeness of the PD group. Thus, the sample consists of 81 adults (*M*_age_ = 35.90; *SD*_age_ = 14.80; 64.2% women) diagnosed with PD (*n* = 39) and from the community (*n* = 42). A combination of both PD and community samples was used to ensure sufficient variability in ToM and personality variables.

DSM-5 categorical PD diagnoses were retrieved from patients’ files and were based on exhaustive professional assessment, including questionnaires and unstructured interviews. Diagnoses included borderline PD (*n* = 15), narcissistic PD (*n* = 7), schizoid PD (*n* = 1), mixed PD (*n* = 12), and other specified PD (*n* = 3). As for medication, 74.36% of the PD participants were taking antidepressants at the time of the study, 48.72% were taking antipsychotics, 5.13% were taking anxiolytics, 7.69% were not taking any medication, and 66.67% were taking two or more medication. Diagnoses and medication information were not available for the community sample. Descriptive and demographic statistics for each group are presented in Table 1.

**Table 1:** Demographic characteristics and mean comparison between the personality disorder sample and the community sample.

Variables	Total sample *n* = 81		Personality disorders *n* = 39		Community sample *n* = 42			
	*M*	*SD*	*M*	*SD*	*M*	*SD*	*t*	*d*
Age	35.90	14.80	35.62	13.36	36.17	16.18	0.166	0.04
Education (years)	16.60	3.43	15.69	3.97	17.48	2.56	2.364*	0.54
Estimated intellectual quotient^a^	102.76	10.80	99.57	10.35	105.78	10.48	2.531*	0.60
	N	%	N	%	N	%	*χ^2^*	Cramer’s V
Gender							3.379	0.20
Man	29	35.8	10	25.6	19	45.2		
Woman	52	64.2	29	74.4	23	54.8		
Occupational status^b^							27.143**	0.58
Employed or retired	33	40.7	15	38.5	18	43.9		
Student	27	33.3	5	12.8	22	53.7		
Unemployed or sick leave	20	24.6	19	48.7	1	2.4		
Marital status							1.046	0.11
Single	58	71.6	30	76.9	28	66.7		
Married/in relationship	23	28.4	9	23.1	14	33.3		

*Notes.* ^a^Estimated IQ ranged from 72 to 125. ^b^Data is missing for one participant. *p < 0.05. **p < 0.001.

All participants completed the ToM tests (as well as other measures) during a meeting with a research assistant. As a compensation, they received $40 CAD. For the self-reported questionnaires assessing personality, 63 participants answered them during the same session. Some participants (18) took part in an earlier phase of the project studying social cognition and had not answered the personality questionnaires. They were contacted in a second step to complete an online version of the questionnaires (due to pandemic restrictions) via the LimeSurvey platform. An additional $50 CAD Amazon gift card was drawn among those participants. The project was approved by the Comité d’éthique de la recherche sectoriel en neurosciences et santé mentale of the Centre intégré universitaire de santé et de services sociaux de la Capitale-Nationale.

### Instruments

#### Theory of mind

The *Combined Stories Test* (COST; 34, 35) was used to assess ToM. The COST is a story-based test that contains items adapted from different well-known ToM instruments, including the *Hinting Task* (34), the *False Belief Task* (35, 36), the *Faux Pas Test* (37), and the *Strange Stories Test* (38). Descriptions of the different types of stories and questions are presented in Supplementary Table 2. Each story, read aloud by the participants, depicts at least two characters in specific situations and is followed by open questions assessing a wide range of mental states, such as beliefs, intentions, or desires. The text remains in front of the participant, who can refer to it as needed. The COST includes 26 ToM questions rated 0 to 2 points according to a validated correction grid (39, 40) for a maximum score of 52 points. A higher score indicates better ToM abilities. The COST also includes different control questions (questions assessing non-social reasoning, elementary ToM skills, or concerning details of the story) that were not targeted for the current study. The test has good internal consistency and excellent test–retest and inter-rater reliability (39, 40). The Cronbach alpha (α) for the present sample is 0.60, and interrater reliability between two members of the research team (ML and AB) assessed with an intraclass correlation coefficient proved to be excellent [*r* (40) = 0.95, *p* < 0.001]. While the COST is typically used as a single global ToM score, the choice was made to compute separate subscores by combining the items inspired by the various tasks composing the COST, similarly to what was previously done by Achim et al. (39; See Supplementary Table 2 for a description). This strategy has the potential to lead to more specific ToM profiles and, thus, to more precise insight into the interrelationships between personality and ToM.

The *Reading the Mind in the Eyes Test* (41) is an extensively used test assessing decontextualized ToM (i.e., participants must make attributions based on complex stimuli without additional contextual information). A French translation of the version revised by Pinkham et al. (42), which provides definitions to minimize reliance on vocabulary skills, was used in the study. The test comprises 36 gray-scale pictures of the eye region presented to the participants on a computer. Participants must identify the mental state represented in the picture from a list of four response choices (three distractors and one good answer). A higher score indicates better ToM abilities, with a maximum of 36 points (one point per correct answer). The RMET has good test–retest reliability with no practice effect (42). Besides having good convergent validity, it has been found to discriminate adequately between healthy controls and patients with schizophrenia (42). The Cronbach alpha for the present sample was relatively low (α = 0.49), which is, however, common with this instrument (43). Nevertheless, the decision was made to use the RMET to ensure continuity with the studies of Fossati et al. and da Costa et al. (26).

#### Personality

The French version of the *Self and Interpersonal Functioning Scale* SIFS (44) is a 24-item self-reported questionnaire assessing AMPD Criterion A. It provides a global personality dysfunction score (α = 0.89), as well as scores for each element: Identity (α = 0.70), Self-direction (α = 0.72), Empathy (α = 0.66), and Intimacy (α = 0.83). A higher score indicates a more severe level of personality dysfunction. The scale has proven useful in distinguishing different degrees of personality pathology based on the ICD-11 (45) and discriminating between clinical and nonclinical groups (44). It also presents good reliability and validity (44, 46).

The validated French version of the *Faceted Brief Form of the Personality Inventory for DSM-5* (PID-5-FBF; 47–49) is a 100-question, self-reported measure that assesses 25 facets of maladaptive personality described in the AMPD (Criterion B), regrouped into five domains. The PID-5-FBF was derived by item-response theory from the original 220-item Personality Inventory for DSM-5. The short form is known to have good reliability and validity [for a review, see (50)]. In the present sample, the Cronbach alphas of the 25 facets ranged from α = 0.76 (Irresponsibility) to α = 0.93 (Anhedonia).

#### Intelligence quotient estimate

The *Matrix Reasoning* and the *Vocabulary* subtests of the *Wechsler Adult Intelligence Scale-IV* (WAIS-IV; 51) were used to estimate the Intelligence Quotient (IQ) with the procedure described by Denney et al. (52) to assess study eligibility.

### Statistical analyses

PD and community samples were combined to perform the cluster analyses for a total of 81 participants. Subscores of the different categories of COST items (see Supplementary Table 2) and the global score of the RMET were computed to be used as clustering variables and standardized before cluster formation. Using six clustering variables falls well within the guidelines prescribed by Formann (53), cited by Sarsted and Mooi (54), regarding the sample size where n = 2^m^, with m being the number of clustering variables. AMPD Criterion A (Identity, Self-direction, Empathy, and Intimacy), the 25 facets composing Criterion B, and sociodemographic variables were used as exogenous variables.

Before performing the primary analyses, all clustering and exogenous variables were screened to ensure that the assumptions of multicollinearity and normality were met. Using correlation coefficients over 0.70 and a variance inflation factor (VIF) < 5 as criteria, no variables showed evidence of multicollinearity (55). According to the kurtosis and skewness coefficients, some variables were not normally distributed and were, therefore, normalized with Templetone’s (56) two-step approach. Nevertheless, even with normalized data, the distribution of the False Belief subscore still presented a ceiling effect, which is expected for this variable corresponding to only two items on the test. Some personality facets also showed floor or ceiling effects when looking at histograms. This could reflect the severity of the PD sample, in which patients endorse very high levels of some personality facets, or a weak endorsement of pathological traits by participants from the community sample. Then, as descriptive analyses, comparisons between PD and community groups on sociodemographic and IQ variables were computed using chi-square (*X*^2^) and *t-test* analyses (see Table 1). The associations between ToM and exogenous variables (sociodemographic and AMPD Criteria A and B personality variables) were also assessed using bivariate Pearson correlations separately in the PD and community samples.

Cluster analyses were computed following a two-stage procedure to determine the presence of representative profiles of ToM abilities. In the first «exploratory stage,» cluster analyses were performed on the six ToM variables with a hierarchical agglomerative clustering procedure to determine possible cluster solutions. The clusters were formed using the Ward method, and the Euclidian distance was used as a measure of similarity. The four possible cluster solutions obtained (ranging from two to five clusters) were compared on visual indicators such as the dendrogram and a scree plot drawn from the agglomeration schedule (54). According to the procedure proposed by Sarstedt and Mooi (54), the variance ratio criteria (VRC; 57) was also computed for every cluster solution. Then, an indicator labeled “ω” was calculated from the VRC to be compared, with a smaller “ω” denoting a better fit to the data.

In the second “confirmatory stage,” the possible cluster solutions identified in the first stage with the visual and the “ω” indicators were compared to select the most fitting model. Thus, TwoStep cluster analyses were performed, forcing a predefined number of clusters according to the solutions retained in stage 1. The TwoStep method also allows seeing which variables weighted the most in the differentiation of the clusters, which is helpful in further interpreting the results. The analyses were performed with the Log-likelihood as a distance measure. The optimal cluster solution was then selected based on the silhouette measure of cohesion and separation, the intercluster differences between the clustering variables, and the interpretability of the results.

Then, the clusters of the selected solution were compared on endogenous (ToM) and exogenous (personality [Criteria A and B] and sociodemographic) variables using chi-square (*X*^2^) tests for categorical variables and Multivariate analyses of variance (MANOVA) for continuous variables. In addition to intercluster comparisons, and since no normative data are currently available, the COST subscores were compared with scores previously obtained by community participants in Achim et al. (39). Thus, a score lower than that of the control group by more than 1.5 standard deviations is considered indicative of ToM difficulties. For example, the study’s control group by Achim et al. (39) obtained a 10.1 score on the Strange Stories questions, with a standard deviation of 1.6. Thus, a score of 8.50 or less will be considered indicative of deficits in the Strange Stories questions. The same approach was used for the RMET, with scores compared with results from the control group of the Social Cognition Psychometric Evaluation (SCOPE) study (42). As for personality variables, the Criterion A severity was estimated in comparison with the study by Gamache et al. (45), and the scores obtained on Criterion B facets obtained in the present study were compared to those of a PD group included in the study by Leclerc et al. (48).

Finally, Cramer’s *Vs* were computed as inter-cluster effect sizes on categorical variables, with Cramer’s V = 0.10 indicating a small effect size, 0.30 indicating a medium effect size, and 0.50 indicating a large effect size (58). Eta squared (η^2^) were computed as global inter-cluster effect sizes for continuous variables and interpreted as follows: η^2^ = 0.01 indicates a small effect size; η^2^ = 0.06 indicates a medium effect size; and η^2^ = 0.14 indicates a large effect size. Additional Cohen’s *d*s were calculated to assess effect sizes on clustering and exogenous variables between each cluster and interpreted according to Cohen’s (58) guidelines (small: *d* = 0.2; medium: *d* = 0.5; large: *d* = 0.8). All statistical analyses were performed using the IBM Statistical Package for Social Sciences Statistics (version 27).

## Results

As presented in Table 1, participants in the PD group presented lower estimated IQ (*d* = 0.60), fewer years of education (*d* = 0.54), and were more likely to be unemployed than the community group. There were no other statistically significant differences between groups on demographic variables. Correlations between ToM tests and estimated intelligence quotient, demographic variables, and personality variables were computed separately for the PD and the Community samples and are presented in Supplementary Tables 3 and 4, respectively.

### Clustering procedure

A hierarchical agglomerative cluster analysis was conducted for the first stage, and solutions ranging from two to five clusters were examined (see Table 2). The visual indices and the VRC were then examined to determine the best-fitting solution. The dendrogram favored the three or five-cluster solutions, while the elbow graph suggested a three-cluster solution for optimal data grouping. The five-cluster solution appeared to be the best-fitting solution when comparing the “ω” indicator derived from the VRC.

**Table 2:** Summary of indicators used to select a cluster solution.

Indicators	Two-cluster solution	Three-cluster solution	Four-cluster solution^2^	Five-cluster solution
Stage 1				
Dendrogram		♦		♦
Scree plot		♦		
ω^1^		187.89	81.49	16.72
Stage 2				
Silhouette measure of cohesion and separation^3^	0.3	0.4	–	0.3
Intercluster contrast		♦	–	♦
Interpretability			–	♦

*Notes.* The symbol ♦ indicates the best-fitting solution for this indicator.

1 The solution with the smallest ω has the best data fit. The ω indicator cannot be computed for a two-cluster solution.

2 The four-cluster solution was not tested in the second stage since no indicators suggested it was the best-fitting solution.

3 A measure of silhouette between 0.2 and 0.5 represents a fair solution (54).

TwoStep cluster analyses were then performed for the confirmatory stage with the two solutions previously retained, i.e., the three and the five-clusters solutions. Both solutions obtained a fair silhouette measure of cohesion and separation (0.4 and 0.3, respectively). The corresponding clusters from the three- and five-cluster solutions were examined to assess which allowed a better distinction between the clusters on ToM variables (intercluster contrast). The interpretability of the cluster solutions was evaluated, considering the demographic and personality variables. Although the three-cluster solution had a slightly higher silhouette measure of cohesion and separation, the five-cluster solution was retained, considering the superior quality of the contrast between the clusters and the more meaningful interpretation it allowed.

### Intercluster comparisons on clustering variables

**Figure 1:**
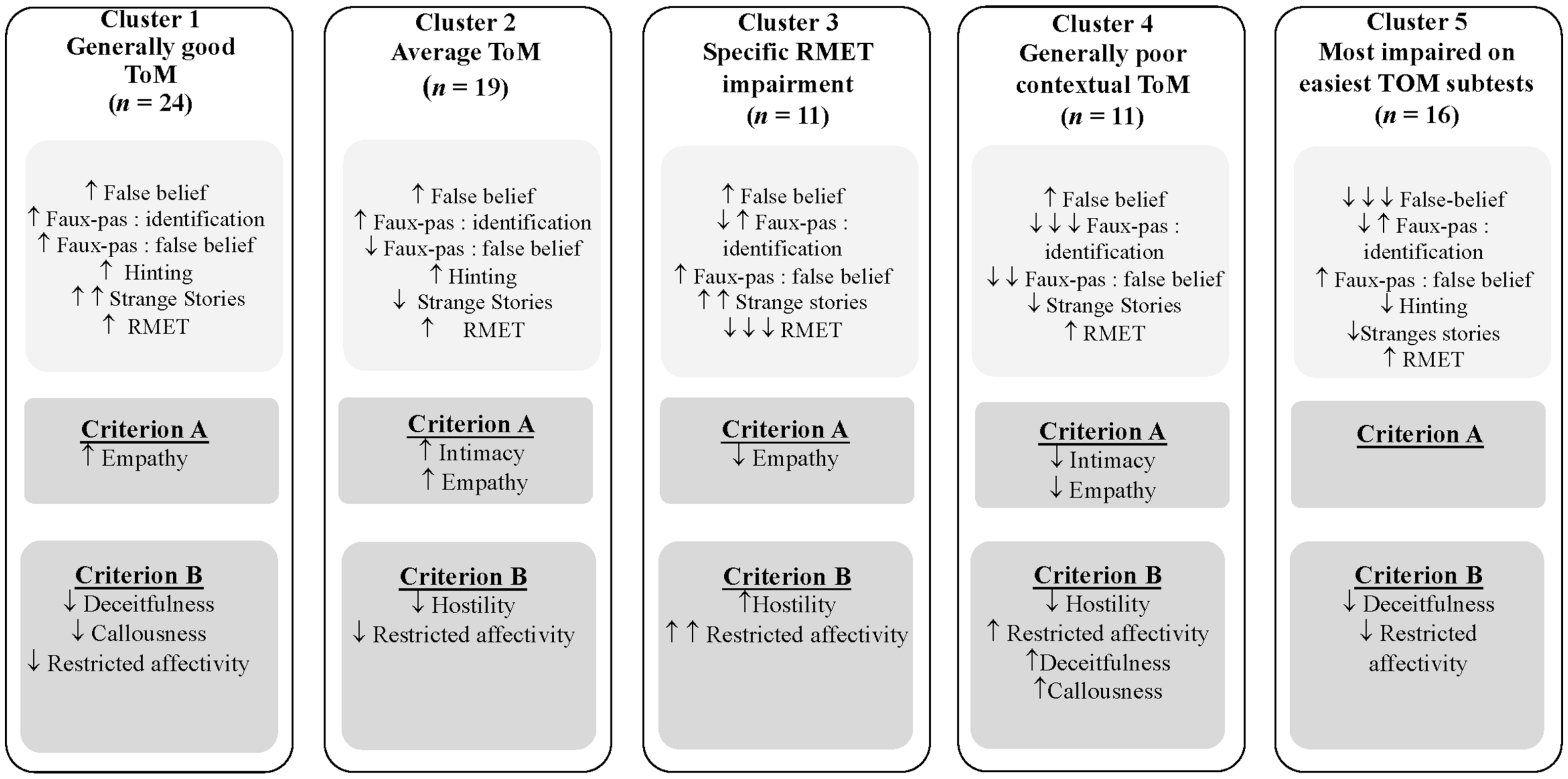
Final five clusters, based on results from ToM tests, and mean comparison analysis on Criterion A and B of the AMPD. Notes. ↑ or ↓ indicates statistical differences with one or two clusters; ↑↑ or ↓↓ indicates statistical differences with three clusters; ↑↑↑ or ↓↓↓ indicates statistical differences with four clusters; RMET =Reading the Mind in the Eyes Test.

Multivariate analyses of variance were conducted to assess if clustering variables significantly differed across the clusters. Significant differences were obtained on all clustering variables (see Table 3 for results of mean comparisons analyses), and a summary of the inter-cluster comparisons is shown in Figure 1. *Post-hoc* tests indicated that individuals from Cluster 1 demonstrated similar or better ToM skills on every score and subscore. Individuals from Cluster 4 and Cluster 5 showed more difficulty on several ToM variables, with Cluster 4 participants scoring lower on both types of questions of the Faux Pas stories, while Cluster 5 was the only one to show deficits on the False Belief questions. Cluster 2 showed average scores on most ToM variables, with lower scores on the False Belief questions of the Faux Pas stories and the Strange Stories questions than Clusters 1 and 3. Finally, participants from Cluster 3 distinguished themselves by having the lower scores on the RMET.

**Table 3:** Results of mean comparisons on demographic, theory of mind, and personality variables (criterion A and B) by clusters.

Variables	1. Generally good ToM *n* = 24		2. Average ToM *n* = 19		3. Specific RMET impairment *n* = 11		4. Generally poor contextual ToM *n* = 11		5. Impaired on easiest TOM subtests *n* = 16				
	*M*	*SD*	*M*	*SD*	*M*	*SD*	*M*	*SD*	*M*	*SD*	*F*	η^2^	*Post-hoc*
Social cognitive variables													
False Belief /4	4.00_a_	0.00	4.00_a_	0.00	4.00_a_	0.00	3.82_a_	0.60	1.88_b_	0.50	145.63***	0.89	5 < 1,2,3,4
Faux Pas: Identification questions /12	10.54_a_	1.14	9.95_a,b_	1.55	8.27_c_	1.56	5.09_d_	1.04	8.44_b_	2.78	21.45***	0.53	4 < 1,2,3,5 3,5 < 1
Faux Pas: False Belief questions /12	11.58_a_	0.83	9.37_b,c_	1.64	11.27_a_	1.01	8.73_b_	1.35	10.50_c_	1.55	13.76***	0.42	4 < 1,3,5 2 < 1,3
Hinting /12	11.04_a_	1.12	11.11_a_	0.88	10.73_a_	0.47	10.09_a,b_	1.81	9.50_b_	1.86	4.67**	0.20	5 < 1,2
Strange Stories /12	10.92_a_	0.88	8.79_b_	1.65	11.18_a_	0.60	8.73_b_	1.79	9.50_b_	1.46	11.83***	0.38	2,4,5 < 1,3
Reading the mind in the eyes /36	27.96_a_	2.81	25.90_a_	2.62	21.56_b_	2.54	26.90_a_	2.43	26.38_a_	4.00	9.10***	0.32	3 < 1,2,4,5
Criterion A^a^													
Identity	2.05	1.02	1.95	0.82	2.40	0.71	2.01	0.78	2.18	0.86	0.53	0.03	
Self-direction	1.39	0.91	1.27	0.85	1.76	0.95	1.88	0.84	1.35	0.76	1.27	0.06	
Empathy	1.15_a_	0.74	0.84_a_	0.71	1.74_b_	0.65	1.79_b_	0.75	1.32	0.66	4.86**	0.21	4 > 1, 2 3 > 2
Intimacy	1.10_a_	0.91	0.86_a,b_	0.74	1.77_c_	1.01	1.96_d_	1.18	1.39	1.03	3.11*	0.14	4 > 2
Criterion B—facets													
Emotional lability	1.23	1.10	1.30	1.05	1.75_a_	0.96	0.93_b_	1.00	1.39	1.09	0.89	0.05	
Anxiousness	1.57	1.06	1.14	1.03	2.09_a_	0.68	1.07_b_	0.99	1.73	0.99	1.79	0.09	
Separation insecurity	1.08	0.93	0.75_a_	0.74	1.43_b_	0.88	0.86	0.91	0.97	0.96	1.15	0.06	
Submissiveness	1.36	0.70	1.08_a_	0.61	1.75 _b_	0.92	1.27	0.95	1.08	0.81	1.68	0.08	
Hostility	0.82_a_	0.77	0.76_a_	0.71	1.68_b_	1.01	0.66_a_	0.92	1.14	0.98	2.88*	0.13	3 > 4, 2
Perseveration	1.12	1.90	0.88_a_	0.65	1.48_b_	0.72	0.98	0.76	1.19	0.95	1.03	0.05	
Depressivity	0.96	1.06	0.55_a_	1.70	1.30_b_	1.05	0.91	1.03	0.89	1.00	1.09	0.05	
Suspiciousness	0.64	0.80	0.49_a_	0.55	1.23_b_	0.79	0.71	0.71	0.77	0.73	1.96	0.09	
Withdrawal	0.93	0.69	0.71_a_	0.68	1.36_b_	0.94	0.98	0.76	1.25	0.97	1.64	0.08	
Anhedonia	1.13	1.14	0.72_a_	0.72	1.52_b_	0.96	1.21	1.13	1.13	0.94	1.22	0.06	
Intimacy avoidance	0.68	0.69	0.49	0.54	0.96	1.11	1.02	0.88	0.92	0.94	1.22	0.06	
Restricted affectivity	0.90_a_	0.60	0.59_a_	0.47	1.64_b_	0.83	1.61_b_	1.07	0.84_a_	0.78	5.91***	0.24	3 > 1,2,5 4 > 2
Manipulativeness	0.55	0.55	0.76	0.73	0.96_a_	0.85	1.05_a_	1.09	0.38_b_	0.50	2.08	0.10	
Deceitfulness	0.38_a_	0.50	0.48_a_	0.43	0.75	0.75	0.97_b_	0.67	0.33_a_	0.49	3.25*	0.15	4 > 1,5
Grandiosity	0.28	0.41	0.52	0.51	0.45	0.42	0.53	0.62	0.22	0.37	1.55	0.08	
Attention seeking	0.88	0.68	1.00	0.87	1.27	0.93	1.16	0.98	0.77	0.81	0.83	0.04	
Callousness	0.29_a_	0.44	0.41	0.50	0.56	0.50	0.90_b_	0.81	0.42	0.44	2.77*	0.13	4 > 1
Irresponsability	0.59	0.66	0.57	0.73	0.98	0.85	1.05	0.76	0.61	0.55	1.49	0.07	
Impulsivity	0.89_a_	0.85	1.05	0.84	1.57_b_	0.61	1.18	1.01	1.05	0.99	1.21	0.06	
Distractibility	1.26	0.97	1.16	0.71	1.71	1.02	1.27	1.15	1.58	1.18	0.80	0.04	
Risk taking	0.85	0.80	0.79	0.81	1.02	0.64	1.39	1.11	0.81	0.73	1.16	0.06	
Rigid perfectionism (lack of)	1.14	0.91	1.00_a_	0.67	1.71_b_	0.46	1.21	0.99	1.08_a_	0.87	1.46	0.07	
Unusual beliefs and experiences	0.37	0.59	0.62	0.87	0.39	0.6	0.59	0.80	0.44	0.63	0.47	0.02	
Eccentricity	0.94	0.95	0.92	0.87	1.39	0.94	0.14	0.88	0.89	1.00	0.64	0.03	
Cognitive and perceptual dysregulation	0.44	0.51	0.53	0.59	0.57	0.60	0.46	0.61	0.37	0.59	0.26	0.01	
Demographic variables													
Age	33.21	15.13	33.84	13.66	30.46_a_	13.24	40.18	17.96	43.19_b_	12.35	1.95	0.11	
Education (years)	16.57	2.04	16.92	4.62	16.23	3.37	17.09	2.98	16.19	4.10	0.18	0.95	
Estimated intellectual quotient	106.35	11.78	104.32	9.36	103.38	5.32	97.78	8.79	97.23	12.50	2.21	0.08	
	N	%	N	%	N	%	N	%	N	%	*χ^2^*	Cramer’s V	
Gender											3.43	0.28	
Man	7	29.2	4	21.1	7	63.6	5	45.5	6	37.5			
Woman	17	70.8	15	78.9	4	36.4	6	54.5	10	62.5			
Occupational status											14.36	0.30	
Employed or retired	7	29.2	8	42.1	3	30.0	6	54.4	9	56.3			
Students	14	58.3	7	36.8	3	30.0	1	9.1	2	12.5			
Unemployed or sick leave	3	12.5	4	21.1	4	40.0	4	36.4	5	31.3			
Marital status											0.84	0.10	
Single	18	75.0	14	73,7	8	72.7	8	72.7	10	62.6			
Married/in relationship	6	25.0	5	26.3	3	27.3	3	27.3	6	37.6			
Group											5.87	0.27	
Personality disorders	9	37.5	7	36.8	7	63.6	5	45.5	11	68.8			
Community	15	62.5	12	63.2	4	36.4	6	54.5	5	31.3			

*Note.* Means with different subscripts (a, b, c) in a same row indicate a difference with a large effect size (Cohen’s *d* > 0.8). *p < 0.05. **p < 0.01. ***p < 0.001.

a A higher SIFS score indicates more severe impairments.

### Intercluster comparisons on exogenous variables

Multivariate analyses of variance were conducted on the AMPD Criteria A and B and demographic variables. The analyses showed no statistically significant differences across clusters on the demographic variables, with small-to-medium effect sizes (see Table 3 for results of mean comparison analyses and Figure 1 for a summary of statistically significant differences). Statistically significant differences were found for Empathy, Intimacy (AMPD Criterion A), Hostility, Restricted affectivity, Deceitfulness, and Callousness (AMPD Criterion B). As summarized in Figure 1, the Post-hoc tests indicated that individuals from Clusters 1 and 2 reported experiencing significantly fewer impairments and pathological personality traits than the other clusters. Participants belonging to Cluster 4 reported experiencing more interpersonal difficulties (Empathy and Intimacy) and antagonistic traits (Deceitfulness and Callousness) but less Hostility and Restricted affectivity than other clusters. Individuals in the third cluster reported higher levels of Hostility than the second and fourth clusters and higher levels of Restricted affectivity than the first, second, and fifth clusters. Participants in the fifth cluster reported significantly lower levels of Deceitfulness and Callousness compared to the fourth cluster and lower levels of Restricted affectivity compared to the third cluster.

## Discussion

The present study aimed to assess whether individuals from clinical (PD) and community samples with distinct ToM profiles exhibit specific personality patterns. To achieve this, clusters were formed based on different scores and subscores of ToM tests. Afterward, the clusters were compared on demographic variables, their respective level of personality functioning, and their score on pathological personality facets. Intimacy and Empathy dysfunction (AMPD Criterion A), as well as multiple antagonistic traits (Deceitfulness, Callousness, Hostility) and Restricted affectivity (AMPD Criterion B), showed the most prominent differences across clusters.

### Correlations

Correlations between ToM tests and personality variables (criteria A and B) were computed. Results for the community sample indicated that a higher level of pathological personality traits is associated with poorer ToM abilities. On the contrary, for the PD group, higher levels of pathological traits are associated with better ToM scores, which may seem counterintuitive. Nevertheless, several studies have shown unimpaired or even enhanced ToM abilities in PD compared to healthy controls (59, 60). Moreover, ToM difficulties in PD have consistently been linked to excessive mental state attributions in BPD (61) and, more recently, to personality pathology in general (62). Thus, interpersonal difficulties in PD would be attributable to an interpretation of mental states that goes beyond social cues rather than to a lack of ToM capacities, which could be attributable to a hypervigilance developed in unstable or unpredictable environments.

Also, despite the theoretical overlap between these two concepts, the SIFS Empathy scale is not significantly correlated with ToM tests. However, numerous studies have shown a lack of association between self-reported empathy scales and objective ToM behavioral tasks (63). Indeed, Murphy et al. (63) reported that scores on self-reported empathy scales accounted for only 1% of the variance on behavioral tests. This may indicate that individuals are not good at evaluating their own empathic abilities. It is also possible that self-reported questionnaires are a more accurate measure of the motivation to empathize, which does not necessarily reflect people’s actual ability to do so, unlike behavioral tests.

### First cluster—generally good ToM

Students represent 58.3% of this cluster, which comprises 62.5% of individuals from the community sample. Individuals in this cluster did not demonstrate any ToM difficulties compared to the other clusters. Instead, they presented a significantly higher ability to infer the meaning of indirect speech than individuals in cluster 5, to understand irony, white lies, or misunderstanding than individuals in cluster 2, 4 and 5, to recognize a faux pas and reason about why it was awkward compared to the individuals in cluster 3, 4 and 5, and to assess if the character possessed the knowledge to infer that s/he was making a faux pas in comparison to individuals in clusters 2 and 4. All these differences had large effect sizes. Finally, because participants presented similar or higher scores on every subscore of the COST than the control group from the Achim et al. (39) study, which indicates good ToM performance, it was labeled *Generally Good ToM*. Interestingly, participants in the PD group, in whom ToM deficits would be expected, represent 37.5% of the individuals in this cluster. Nevertheless, it has previously been shown that ToM deficits in PDs are more easily evidenced in more complex tasks (e.g., tasks with stimuli integrating several modalities like videos) or tasks or contexts more emotionally charged (61, 62). Thus, it remains conceivable that these participants present ToM impairments that would be highlighted in more complex or emotionally arousing tasks. However, by identifying that a certain proportion of the PD group performed very well on these ToM tasks and was therefore included in *the Generally Good ToM* Cluster, the cluster analyses approach of the present study highlighted a high variability in the ToM skills of this population. This calls for more studies assessing individual differences in ToM skills among the PD population.

The only Criterion A element for which the individuals in this cluster differed from the other clusters was Empathy; they reported less difficulty empathizing than individuals from clusters 3, 4, and 5. According to the study by Gamache et al. (45), their level of Empathy is comparable to that of individuals with mild PD. Furthermore, they did not exhibit a high level of any pathological personality facets when compared with the PD sample from the Leclerc et al. study (48). Instead, they reported experiencing less Restricted affectivity than Cluster 3 and described themselves as more honest, genuine, and concerned about others (lower Callousness and Deceitfulness) than Cluster 4. This description is similar to the definition of Agreeableness, which has been positively associated with ToM skills (5–7). Agreeable individuals are more prone to exhibit prosocial behaviors (64) and tend to have good social functioning and more satisfying relationships (65). These results suggest this could be attributed to a better ability to understand what others are thinking or feeling (ToM tests and Empathy).

### Second cluster—average ToM

The second cluster comprises 63.2% of community participants. This cluster is characterized by poorer performance on questions assessing the ability to interpret irony, misunderstanding, or white lies and the ability to infer the knowledge a character had before he committed a faux pas when compared to the *Generally Good ToM* cluster and the Cluster 3, but with a better capacity to understand the true meaning behind indirect demands than the Cluster 5. While none of the ToM scores reached the 1.5 standard deviation cut-offs established to indicate deficits, all differences showed large effect sizes. Furthermore, the average score on the Strange Stories questions was lower than that of the psychotic group from the Achim et al. (39) study, which suggests a certain level of difficulties. Despite the absence of marked deficits on other ToM tests, they appear less proficient at identifying mental states than the *Generally Good ToM* cluster and were, therefore, called *Average ToM.*

However, despite lower scores than individuals in the *Generally Good ToM* cluster on some ToM variables, they reported having better interpersonal functioning, which is somewhat contradictory. In fact, individuals in this cluster described themselves as more empathetic than the Clusters 3 and 4 participants and more comfortable developing intimate relationships than Cluster 4 participants. Furthermore, according to the study by Gamache et al. (45), individuals in the *Average ToM* cluster reported a level of interpersonal functioning similar to people without personality disorders. In contrast, the *Generally Good ToM* cluster participants reported interpersonal abilities indicative of mild PD.

This may reflect the weak association between self-reported measures of empathy and objective ToM tests (63, 66). It could also be indicative of an overconfidence pattern sometimes observed in PD patients (67) that has been linked to ToM deficits (68), such that individuals with PD tend to be more confident in their responses or interpretation of social situations, even when they are mistaken.

### Third cluster—specific RMET impairment

This cluster mainly comprises male (63.6%) and PD (63.6%) participants. They presented a significantly lower capacity than participants from the other clusters to recognize emotions based on eye gaze, with large effect sizes. Compared with the individuals from the control group of the SCOPE study (42), individuals for this cluster did not exhibit lower scores according to the 1.5 standard deviation criterion, probably because the standard deviation is quite large. However, their score (*M* = 21.56) is almost identical to that of individuals with psychotic disorders from the SCOPE study (*M* = 21.28), who displayed significant differences with the control group of the same study and who are known to display ToM impairments. Thus, individuals from this cluster seem to exhibit some difficulties, even though they do not meet our established criterion of 1.5 standard deviations. As a result, it was labeled *Specific RMET Impairment*. The RMET differs from the other ToM variables since it assesses decontextualized ToM. Therefore, the deficits observed in this group may be more subtle and occur under more specific conditions, such as when participants cannot rely on context to help them understand others’ mental states. However, Etchepare et al. (69) highlighted two profiles of impaired social cognition in a community sample, with one profile showing deficits specifically on facial emotion recognition, as measured notably by the RMET. In fact, the RMET has sometimes been considered an emotion recognition task (70). The marked presence of specific RMET impairments in this cluster compared to the other clusters supports the idea of a distinction between the RMET and other ToM tasks, as well as the existence of a subgroup of individuals presenting impairments specifically when identifying facial emotions.

While showing better abilities to identify irony, misunderstanding, or white lies than Cluster 4, this cluster also exhibited other impairments, albeit less pronounced than the RMET deficits but still with large effect sizes. They showed more difficulty than the *Generally Good ToM* cluster when they had to identify a faux pas and explain why it was a faux pas (identification questions of the Faux Pas stories), but the score is not indicative of a marked deficit according to the 1.5 standard deviation criterion when comparing with the scores of participants from the study by Achim et al. (39)

In contrast, they demonstrated capacities to understand that a faux pas comes from an incorrect belief on the part of the person who committed it (False Belief questions of the Faux Pas stories) comparable to the *Generally Good ToM*. They even exhibited superior capacity than both the *Average ToM* and fourth clusters and a higher score than the control group of Achim et al. (39) group on these questions.

Thus, they seem more skilled in answering the False Beliefs questions than the identification questions of the Faux Pas stories. This discrepancy between the results of the two types of questions in the Faux Pas stories may be surprising. However, several authors have suggested that the *Faux Pas Task* measures two distinct traits of ToM: affective ToM (the ability to infer emotions) and cognitive ToM (the ability to infer other’s intentions or beliefs; 71). Thus, the identification questions of the Faux Pas stories, which require taking into account the emotional impact of the faux pas, would assess affective ToM. In contrast, the False-Belief questions of the Faux Pas stories would assess cognitive ToM. According to the classification of Németh et al. (71), the RMET is also a task assessing affective ToM, while the other COST questions would assess cognitive ToM. Therefore, individuals in this cluster seem to infer cognitive mental state more easily than emotions (affective ToM). This could be partly explained by the large proportion of men in this cluster. Indeed, it is well documented that men experience and express their emotions less intensely and are less skilled at recognizing emotions than women (72). This could have implications for therapeutic treatment, suggesting that interventions targeting the ability to recognize emotions may benefit men seeking psychological help. These results are also consistent with those of da Costa et al. (26), who found that sex could moderate the relationship between ToM and the RMET.

Furthermore, individuals in this cluster presented statistically significant inferior self-reported empathic abilities compared to the *Average ToM* cluster, at a level representative of a moderate PD, according to Gamache et al. (45). They also reported experiencing a more limited range of emotions (Restricted Affectivity) than the *Generally Good ToM*, *Average ToM* and fifth clusters, with nevertheless a higher propensity to experience anger (Hostility) than individuals in the *Average ToM* and the fourth cluster. Scores on both facets are comparable with scores of outpatients with PD (48). It has been documented that the ability to interpret the emotions of others is linked to the ability to reflect on one’s own feelings and that common neural substrates underly these two abilities (73). Thus, the cooccurrence of difficulty reflecting on their own emotions (Restricted affectivity) and those of others (ToM deficits) present in this cluster is coherent. The higher level of Hostility may reflect a greater severity of personality impairment since almost two-thirds of individuals in this cluster came from the PD group. Finally, with difficulties in inferring emotions in others, their interpersonal relationships may be more unpredictable or conflictual, which would be reflected in their reported anger.

### Fourth cluster—generally poor contextual ToM

Individuals from the fourth cluster, comprising 45.5% of PD participants, were less skilled in detecting irony, white lies, or misunderstanding than those forming the *Generally Good ToM* and the *Specific RMET Impairment* clusters. Furthermore, their score on these questions was closer to the score from the psychotic group than the control group from the study by Achim et al. (39). They also performed more poorly than all of the other clusters when they had to detect a faux pas or to reason about the knowledge of the individual making the faux pas, except when compared to the *Average ToM* cluster. All effect sizes were large. Nevertheless, only the score of the identification questions was indicative of impairments according to our 1.5 standard deviation criterion. Since they exhibited substantial deficits in ToM abilities compared to the other clusters, it was labeled *Generally Poor Contextual ToM*.

In addition to impairments in ToM skills, participants from this cluster reported being less empathetic than those from the *Generally Good ToM* and *Average ToM* clusters, comparable to people with moderate PD. They also reported more intimacy struggles than individuals from the *Average ToM* cluster, to the extent of individuals with moderate PD (45). The results partially confirm our hypothesis that more pronounced impairments in the interpersonal functioning scale of Criterion A would be found in clusters exhibiting poorer ToM performance, since the definition of Empathy partly overlaps with that of ToM and that a good capacity to understand the experience of others and the impact of our behaviors on them naturally favors the development of intimate relationships. Nevertheless, correlations between Empathy and ToM tests were not statistically significant.

Individuals from this cluster also described themselves as having little regard for the feelings or well-being of others (high level of Callousness), being dishonest or untruthful (high level of Deceitfulness), and showing diminished emotional response (Restricted affectivity), especially regarding guilt (Callousness). In fact, their scores on these three facets are higher than the scores of PD patients (48). Antagonistic traits have been associated with lower relationship satisfaction, with the use of strategies or actions that may be harmful to their partners (e.g., infidelity, coercion) and with more intimate partner violence (74). Thus, it is consistent that individuals in clusters with higher levels of antagonistic traits also reported poorer interpersonal abilities (Criterion A).

These results partly confirm our hypothesis that participants exhibiting higher antagonistic traits would perform more poorly on social cognitive tests. Indeed, while individuals in this cluster presented more ToM difficulties than those from other clusters, as well as a pathological level of Callousness and Deceitfulness, they did not report a higher propensity to experience anger, irritability, or vengeful behaviors (i.e., Hostility), which is less characteristic of the Antagonistic domain. However, this low propensity for Hostility is consistent with the indifference and limited emotional responses also representative of this group (Restricted affectivity), which was associated with antagonistic traits in previous works. For example, the concept of primary psychopathy describes individuals who are manipulative, callous, and who show little empathy while being characterized by low levels of anxiety and emotional response (75).

### Fifth cluster—impaired on easiest subtests

The last cluster comprises 68.8% of participants with a PD. Individuals in this cluster presented considerable difficulty understanding that a character has a different knowledge than their own (False Belief), a skill generally acquired in childhood (3). In addition to being the cluster with the lowest score on False Belief stories, with large effect sizes when comparing to all other clusters, their score differed by more than 1.5 standard deviations not only from the control group in the Achim et al. (39) study but also from the group of psychotic patients. They also exhibited weaker abilities than individuals from *the Generally Good ToM* cluster in identifying a faux pas, but with a score comparable to the control group from the study by Achim et al. (39). They also less easily understood irony, misunderstanding, or white lies than individuals from the *Generally Good ToM* and the *Specific RMET Impairment* clusters, in addition to being more challenged than the *Generally Good ToM* or the *Average ToM* clusters when inferring the meaning of indirect speech, with only the latter meeting the 1.5 standard deviation criterion. All these differences showed large effect sizes. These results suggest pervasive ToM impairments in this cluster, thus labeled *Impaired on Easiest Subtests.* Impairments in the False Belief questions were so extensive that we wondered whether cognitive issues might underlie these difficulties since cognitive function is related to ToM (18). Indeed, this cluster had the lowest estimated IQ along with the *Generally Poor Contextual ToM* cluster, but it is still within the normal range. Furthermore, except for the Hinting questions (PD and community), the False Beliefs questions (PD), and the Strange Stories questions (community), the correlations between IQ and the ToM subtests were not significant. Thus, lower cognitive ability does not seem to underlie the errors committed in ToM tests.

In addition, according to Criterion A, individuals in this cluster did not differ from the other clusters but still reported personality functioning comparable to individuals with mild PD (45). They also reported lower Restricted Affectivity than the *Specific RMET Impairment* cluster and described themselves as more honest than individuals in the *Generally Poor Contextual ToM* cluster. These results run counter to our hypothesis that clusters exhibiting ToM deficits would be characterized by elevation of antagonistic or schizotypal personality traits. On the other hand, 68.8% of the group had severe PD, as well as ToM deficits, suggesting they could potentially present more interpersonal difficulties than their results on self-administered questionnaires suggest. Thus, the contrast between results on ToM tests and self-reported instruments may be indicative of a tendency to respond in a way deemed more socially acceptable by attenuating personality traits they consider problematic, to preserve their self-image or to avoid the consequences of the stigma surrounding the diagnosis of PD (76). It could also be due to a lack of insight, i.e., their perception of themselves is distorted. Indeed, it is recognized that people with PD may have difficulty identifying their pathological traits, or their impact on interpersonal functioning (77).

### Schizotypy

Finally, given the significant deficits in ToM skills observed in schizophrenia spectrum disorders, we hypothesized that clusters with poorer performance in ToM tests would exhibit an elevation of the traits that comprise the schizotypal PD, including Unusual beliefs and experiences, Eccentricity, and Cognitive and Perceptual dysregulation of the Psychoticism domain in addition to the Restricted affectivity, Withdrawal, and Suspiciousness facets from the Detachment domain. However, the obtained results did not strongly support this hypothesis. Only the Restricted affectivity trait was found to significantly discriminate among groups. In contrast, none of the Psychoticism domain traits distinguished between clusters, with negligible effect sizes. These results are consistent with previous studies by Fossati et al. (25) and da Costa et al. (26), which found more robust links between ToM and the Detachment domain than the Psychoticism domain.

### Strengths and limitations

This study has several strengths, including the integration of clinical and nonclinical samples and both Criteria A and B of the AMPD in the study of ToM. Nevertheless, several limitations should be addressed.

Firstly, caution is needed when generalizing results. Notably, the presence of a PD or other mental health disorder was not assessed in the community sample, even though, based on the cut-offs established by Gamache et al. (45) for the SIFS, 61.9% of the community sample reported mean scores of personality dysfunction that does not reach the severity of a PD. The lack of control for comorbid conditions calls for careful interpretation when generalizing the results to healthy individuals, especially since psychological studies are known to attract participants with higher levels of depression, anxiety, or PD (78). Further studies should assess whether comorbid mental health disorders could influence the relationship between ToM and personality. Additionally, the overrepresentation of women in the sample (64.2%) raises concerns about generalizing the findings to male populations, and financial compensation may have introduced a selection bias favoring lower-income participants.

Secondly, the study’s sample size, while providing sufficient statistical power to detect effects corresponding to moderate to large effect sizes, was still relatively small. This limitation constrained the number of variables included in cluster formation. Moreover, the personality assessment relied solely on self-reported questionnaires, introducing the potential for response biases. Participants may have portrayed themselves more favorably or may have lacked insight into their personality difficulties.

Finally, the RMET exhibited poor psychometric properties, which has also been previously reported for this instrument. This has prompted some researchers to question its validity. However, its inclusion in this study aimed to facilitate comparison with previous studies exploring the relationship between ToM and AMPD-based personality pathology (25, 26).

### Clinical implications

Despite the aforementioned limitations, the study’s findings can have significant clinical implications. Indeed, individuals with significant impairments in ToM may lack the necessary skills to benefit from psychological therapy addressing personality. Simultaneously, personality traits can pose challenges to interventions, especially those designed to enhance ToM. Therefore, gaining a deeper understanding of which personality traits are associated with specific profiles of ToM impairments could empower clinicians not only to choose the most appropriate treatment but also to anticipate potential obstacles and customize existing approaches to enhance their effectiveness for clients with ToM deficits or pathological personality traits.

## Conclusion

This was the first study to jointly investigate Criteria A and B of the AMPD in relation to ToM. In addition to highlighting different profiles of ToM, we showed that impairments in Intimacy and Empathy (AMPD Criterion A), as well as multiple antagonistic traits (Deceitfulness, Callousness, Hostility) and Restricted affectivity from the Detachment domain (AMPD Criterion B), showed the most significant differences across clusters. This supports the idea that personality traits, Antagonism, and also Detachment—though to a lesser extent—are associated with ToM dysfunctions. However, poorer ToM test performance was not exclusively found in clusters exhibiting high antagonistic traits or impairments in personality functioning. These observations highlight how ToM and personality pathology, two key areas underpinning interpersonal problems, can co-occur in some profiles but are not necessarily isomorphic. It may also suggest a lack of insight in a number of participants when using self-reported measures to probe for personality pathology. Therefore, encouraging the assessment of both constructs, especially in clinical populations, could be the best course of action whenever possible and could positively influence treatment modalities and interventions.

## Data availability statement

The datasets presented in this article are not readily available because of the local ethics committee restrictions. Requests to access the datasets should be directed to claudia.savard@fse.ulaval.ca.

## Ethics statement

The studies involving humans were approved by Comité d’éthique de la recherche sectoriel en neurosciences et santé mentale of the Centre intégré universitaire de santé et de services sociaux de la Capitale-Nationale. The studies were conducted in accordance with the local legislation and institutional requirements. The participants provided their written informed consent to participate in this study.

## Author contributions

ML: Data curation, Formal analysis, Funding acquisition, Investigation, Methodology, Project administration, Software, Writing – original draft, Writing – review & editing. AA: Conceptualization, Funding acquisition, Methodology, Project administration, Resources, Supervision, Writing – review & editing. DG: Supervision, Writing – review & editing. AB: Data curation, Writing – review & editing. SS: Supervision, Writing – review & editing. CS: Conceptualization, Funding acquisition, Investigation, Methodology, Resources, Supervision, Validation, Writing – review & editing.
