# Supplementary material for: Profiles of theory of mind impairments and personality in clinical and community samples: integrating the alternative DSM-5 model for personality disorders
Source: Front Psychiatry. 2024 Jan 11;14:1292680. doi: 10.3389/fpsyt.2023.1292680 (PMC10809153; doi:10.3389/fpsyt.2023.1292680)
Supplement: Supplementary file 1 [file Table_1.pdf]

Supplementary Material

Profiles of theory of mind impairments and personality in clinical and community samples: Integrating the Alternative DSM-5 model for personality disorders

Mireille Lampron*, Amélie M. Achim, Dominick Gamache, Allyson Bernier, Stéphane Sabourin and Claudia Savard

*** Correspondence:**
Corresponding Author: mireille.lampron.1@ulaval.ca

Supplementary Table 1.

Description of Criterion A and B of the Alternative DSM-5 Model for Personality Disorder

Criterion A	Elements description
Identity	Experience of oneself as unique, with clear boundaries between self and others; stability of self-esteem and accuracy of self-appraisal; capacity for, and ability to regulate, a large range of emotional experience.
Self-direction	Pursuit of coherent and meaningful short-term and life goals; utilization of constructive and prosocial internal standards of behavior.
Intimacy	Maintenance of multiple satisfying and enduring relationships; desire and capacity to engage in caring, close, reciprocal, and cooperative relationships.
Empathy	Comprehension and appreciation of others' experiences and motivations; tolerance of differing perspectives; understanding the effects of one's own behavior on others.
Criterion B	Domain's description

Negative Affectivity	Frequent and intense experiences of high levels of a wide range of negative emotions (e.g., anxiety, depression, guilt/shame, worry, anger) and their behavioral (e.g., self-harm) and interpersonal (e.g., dependency) manifestations.
Detachment	Avoidance of socioemotional experience, including both withdrawal from interpersonal interactions (ranging from casual, daily interactions to friendships to intimate relationships) and restricted affective experience and expression, particularly limited hedonic capacity.
Antagonism	Behaviors that put the individual at odds with other people, including an exaggerated sense of self-importance and a concomitant expectation of special treatment, as well as callous antipathy toward others, encompassing both an unawareness of other's needs and feelings and a readiness to use others in the service of self-enhancement.
Disinhibition	Orientation towards immediate gratification, leading to impulsive behavior driven by current thoughts, feelings, and external stimuli, without regard for past learning or consideration of future consequences.
Psychoticism	Exhibiting of a wide range of culturally incongruent odd, eccentric, or unusual behaviors and cognitions, including both process (e.g., perception, dissociation) and content (e.g., beliefs).

Note. Adapted from the American Psychiatric Association, 2013

Supplementary Table 2.

Description of the Item Categories of the Combined Stories Test

Original tasks	Stories
Hinting	The stories feature two characters, one of whom makes an obvious but indirect hint. Participants must deduce what the character really meant.
Faux pas	The stories feature two or more characters, one of whom makes a social faux pas, i.e., saying something awkward or upsetting without realizing it. The participant must detect the faux pas (**identification questions**) and reason about whether the character previously had the knowledge to know he or she was making a mistake (**false belief questions**).
False belief	The stories feature two or more characters with different knowledge. To answer correctly, the participant must understand that the character has a different knowledge than their own.
Strange Stories	The stories present two characters, one of whom makes a statement that cannot be understood without considering an underlying, but not clearly specified, mental state. The stories assess irony, white lies, or misunderstanding.

Supplementary Table 3.

Correlations Between Demographic Variables, Intelligence Quotient, Personality Variables, and Theory of Mind Tests for the Personality Disorders Sample

Variables	*M* (*SD*)	COST total score	False beliefs	Faux pas: Identification	Faux pas: False belief	Hinting	Strange Stories	Reading the Mind in the Eyes
Demographic variables								
Age	35.62 (13.36)	-.32*	-.35*	-.23	-.17	-.01	-.33*	-.20
Education (years)	15.69 (3.97)	.01	.03	-.02	-.02	.35*	-.24	-.13
Estimated intelligence quotient [a]	99.57 (10.35)	.37*	.34*	.18	.20	.59*	.09	-.14
Personality variables								
Criterion A								
Identity	2.70 (.63)	.38*	.33*	.34*	.15	.31	.15	.13
Self-direction	2.01 (.83)	.24	.37*	.24	.02	.04	.10	.07
Empathy	1.69 (.61)	-.02	.20	-.20	-.03	.05	-.07	-.17
Intimacy	1.91 (.95)	-.13	.14	-.11	-.05	-.03	-.25	-.11
Criterion B – facets								
Emotional lability	2.02 (.85)	.49**	.26	.48*	.18	.23	.36*	-.06
Anxiousness	2.26 (.63)	.36*	.14	.35*	.22	.15	.21	.12
Separation insecurity	1.38 (.90)	.25	-.02	.21	.13	.30	.16	.05
Submissiveness	1.62 (.84)	.18	.21	.20	-.05	-.01	.22	-.14
Hostility	1.40 (.86)	.36*	.07	.18	.34*	.19	.35*	-.07
Perseveration	1.56 (.76)	.09*	.03	.29	.19	.11	.53**	.06
Depressivity	1.54 (.93)	.13	.30	.07	.10	.06	-.01	-.18
Suspiciousness	1.13 (.78)	.02	.11	-.07	.13	-.08	.03	-.12
Withdrawal	1.51 (.79)	-.01	-.07	.02	.14	-.09	-.08	-.14
Anhedonia	1.86 (.83)	.08	.25	.02	.12	.14	-.15	-.06
Intimacy avoidance	1.12 (.91)	-.13	-.13	-.13	.07	-.24	-.02	-.20
Restricted affectivity	1.28 (.90)	-.11	.14	-.19	-.04	-.13	-.02	-.26
Manipulativeness	0.74 (.77)	-.04	.29	-.12	-.06	.11	-.17	.01
Deceitfulness	0.63 (.65)	-.07	.15	-.20	-.15	-.04	-.01	.03
Grandiosity	0.35 (.43)	-.08	.08	-.06	-.22	.18	-.17	-.26
Attention seeking	1.02 (.91)	.03	.06	-.03	-.06	.36*	-.14	.11
Callousness	0.06 (.58)	-.26	.13	-.25	-.20	-.12	-.26	-.11
Irresponsability	1.04 (.71)	.07	.28	.02	.01	-.07	-.03	-.04
Impulsivity	1.51 (.90)	.11	.15	.13	-.06	.12	.04	-.02
Distractibility	1.85 (.92)	.26	-.15	.14	.27	.08	.39*	.06
Risk taking	1.28 (.85)	.04	.17	.09	-.06	.14	-.15	.06

Rigid perfectionism (lack of)	1.53 (.76)	.37*	.12	.27	.26	.41*	.14	-.05
Unusual beliefs and experiences	0.72 (.82)	-.20	.04	-.04	-.26	-.01	-.32*	.17
Eccentricity	1.38 (.88)	.22	.30	.32	-.04	.11	.04	.01
Cognitive and perceptual dysregulation	0.70 (.59)	.17	.31	.31	-.14	-.01	.08	-.00

Note. COST = Combined Stories Test; A higher *Self and Interpersonal Functioning Scale* score indicates more severe impairments.

[a] Data for IQ was missing for five participants.

*p < .05, **p < .01.

Supplementary Table 4.

Correlations Between Demographic Variables, Intelligence Quotient, Personality Variables, and Theory of Mind Tests for the Community Sample

Variables	*M (SD)*	COST total score	False belief	Faux pas: Identification	Faux pas: False belief	Hinting	Strange Stories	Reading the Mind in the Eyes
Demographic variables								
Age	36.16 (16.18)	-.18	-.31*	-.03	-.11	.01	-.23	-.07
Education (years)	17.48 (2.56)	.06	-.32*	.14	-.14	.09	.18	-.07
Estimated intelligence quotient [a]	105.78 (10.48)	.53	.08	.48*	.25	.49*	.16	.18
Personality variables								
Criterion A								
Identity	1.56 (.67)	.09	-.10	.12	.04	.03	.06	-.02
Self-direction	1.01 (.59)	-.23	.18	-.40**	-.16	-.01	-.02	.10
Empathy	0.88 (.71)	-.23	-.03	-.30	-.13	-.10	-.01	-.05
Intimacy	0.80 (.74)	-.37*	.00	-.42**	-.21	-.14	-.10	.01
Criterion B - facets								
Emotional lability	0.65 (.75)	.04	.05	.01	-.12	.05	.16	-.11
Anxiousness	0.91 (.84)	.26	.03	.14	.21	.15	.19	-.15
Separation insecurity	0.64 (.72)	.22	.05	.17	.18	.00	.18	-.10
Submissiveness	0.96 (.57)	.20	.17	.13	-.09	.13	.28	.22
Hostility	0.57 (.73)	-.11	-.07	-.11	-.17	.14	-.08	-.20
Perseveration	0.68 (.62)	.02	.08	-.03	-.02	.05	.05	-.01
Depressivity	0.28 (.51)	.11	-.12	.12	-.05	.17	.08	-.17
Suspiciousness	0.33 (.42)	-.02	.07	-.09	-.11	.10	.06	-.08
Withdrawal	0.54 (.48)	.07	-.01	.05	.04	-.04	.08	-.15
Anhedonia	0.39 (.48)	.10	.03	-.01	-.00	.06	.16	-.08
Intimacy avoidance	0.44 (.54)	.04	.13	-.09	.12	-.03	.10	-.12
Restricted affectivity	0.76 (.62)	-.17	.16	-.34*	-.09	.01	.03	.03
Manipulativeness	0.64 (.71)	.02	.13	.07	-.20	.05	.09	-.09
Deceitfulness	0.42 (.49)	-.21	.23	-.30	-.22	-.04	-.07	-.21
Grandiosity	0.41 (.49)	-.26	.16	-.30	-.29	-.04	-.02	.07
Attention seeking	0.93 (.76)	.19	.07	.04	.08	.01	.36*	-.07
Callousness	0.33 (.49)	-.20	.06	-.24	-.13	-.01	-.11	-.02
Irresponsability	0.39 (.54)	-.27	-.01	-.29	-.30	-.09	-.10	.08
Impulsivity	0.70 (.66)	.05	.17	-.10	-.01	.21	.06	.06
Distractibility	0.91 (.84)	.00	.09	-.07	-.12	.00	.19	-.81
Risk taking	0.60 (.64)	-.20	.14	-.28	-.09	-.13	-.01	.12

Rigid perfectionism (lack of)	0.86 (.75)	.00	.10	-.10	-.06	-.02	.18	.03
Unusual beliefs and experiences	0.24 (.45)	-.04	.08	.02	-.26	-.12	.20	.03
Eccentricity	0.67 (.83)	-.08	-.02	-.09	-.05	-.19	.14	-.07
Cognitive and perceptual dysregulation	0.26 (.44)	-.09	.01	-.05	-.18	-.11	.10	.10

Note. COST = Combined Stories Test. A higher *Self and Interpersonal Functioning Scale* score indicates more severe impairments.

[a] Data for IQ was missing for four participants.

*p < .05, **p < .01.
